# Riding a Golf Cart Versus Walking: A Study on the Physiological and Performance Differences in Tournament Golf

**DOI:** 10.1002/ejsc.70099

**Published:** 2025-12-08

**Authors:** Amy O'Donnell, Andrew Murray, Adam Jones, James E. Harrison, Alex Lindsay, Tony Bennett, Chris Bishop, Tim F. Donovan, James P. Morton, Carl Langan‐Evans, Graeme L. Close

**Affiliations:** ^1^ Research Institute for Sport and Exercise Sciences Liverpool John Moores University Liverpool UK; ^2^ Ladies European Tour Buckinghamshire Golf Club Denham Court Drive Denham UK; ^3^ PGA European Tour Health and Performance Institute Virginia Water UK; ^4^ Medicine and Science The R&A St Andrews UK; ^5^ Tournament Golf College Cornwall UK; ^6^ European Disabled Golf Association (Medical and Scientific Department) Wassenaar the Netherlands; ^7^ International Golf Federation (Disability and Inclusion Department) Maison du Sport International Lausanne Switzerland; ^8^ Faculty of Science and Technology London Sport Institute Middlesex University London UK

**Keywords:** cognitive, energy expenditure, equality, golf, golf‐cart, performance

## Abstract

Golf demands sustained physical effort and effective fatigue management, especially in competitive play. Allowing players to ride golf carts in elite play has raised concerns about potential performance advantages, yet well‐controlled studies are lacking. This study examined the effects of golf cart use on physiological, physical and cognitive outcomes in competitive golfers. Sixteen males (mean age: 21 ± 3 years; handicap: 2.3 ± 3.7) completed two randomised competitive rounds on a championship course (6587m; 19°C), either walking with a caddie or riding a golf cart. Physiological measures included activity energy expenditure (Actiheart), core temperature, heart rate and perceived exertion (0–100). Physical outcomes were step count, carry distance, clubhead speed, ball speed and muscle power. Cognitive workload (NASA‐Task Load Index) was assessed post‐round. The step count and activity energy expenditure were significantly higher for walking than using a golf cart (17,007 ± 1708 vs. 6274 ± 1111 steps; 880 ± 279 vs. 456 ± 155 kilocalories). Core temperature was higher for walking at holes 6, 12, and 18 (*p* = 0.022). The heart rate increased across the round when walking but decreased while using a cart (*p* < 0.01), and post‐round exertion was higher for walking (41 ± 19 vs. 25 ± 14 and *p* < 0.001). Carry distance, clubhead and ball speed did not differ. NASA‐Task Load Index subscales of physical demand and performance (reverse scored) were higher for walking. Relative to walking, golf cart use lowered internal physiological and external physical load, without impairing muscle power or shot performance. Cognitively, walking imposed higher physical strain and reduced perceived performance. Further research should explore whether these physiological, physical and cognitive outcomes impact performance across multiday tournaments.

## Highlights

- Walking the golf course substantially increased physiological and physical demands, including energy expenditure, heart rate, core temperature, step count and perceived exertion, compared with riding a golf cart.
- Neuromuscular performance, as indicated by countermovement jump metrics, declined more after walking, whereas golf‐specific performance (clubhead and ball speed) remained unaffected over a single round.
- Perceived performance and cognitive engagement were not impaired by walking, suggesting that higher physical strain does not necessarily reduce subjective or technical performance.
- Golf carts use may mitigate fatigue and preserve neuromuscular function, highlighting practical implications for multi‐round or tournament play, and future research should examine cumulative effects across diverse populations and conditions.

## 1 Introduction

Golf has worldwide reach, with approximately 100 million participants, both amateur and professional engaging in both on‐course and off‐course activities, including driving ranges and golf simulators (The_R&A 2024). Often at the elite level, competitive play is physically demanding where tournament rounds often exceed 10 km and 4–6 h repeated over consecutive days, requiring sustained endurance and resilience (O'Donnell et al. 2024). As a health‐enhancing physical activity, long‐term participation is associated with numerous physical and mental health benefits (Luscombe et al. 2017; Murray et al. 2017).

In contrast, recreational golfers in Europe have traditionally walked the course using trolleys or carrying their bags; the riding of golf carts has become increasingly prevalent, particularly by players with medical conditions or disabilities (Hall et al. 2025). By comparison, elite amateur and professional golfers are prohibited from riding golf carts during most tournaments organised by governing bodies such as The Royal & Ancient (R&A) and the United States Golfing Association (USGA) without a qualifying medical reason. This restriction has renewed debate about the fairness of golf cart use, particularly regarding potential advantages in reducing physical and physiological load and possibly cognitive demands, which could impact competitive fairness.

The assessment of physiological and cognitive demands in golf offers important insights into performance optimisation and fatigue management. Regardless of whether golfers walk or ride a golf cart, strategic adaptations may help preserve endurance, concentration and decision‐making throughout play. However, research examining activity energy expenditure (AEE) and performance outcomes associated with golf cart use remains limited and inconclusive. A recent scoping review (O'Donnell et al. 2024) highlighted wide variability in reported AEE, with estimates ranging from 531 to 2467 kilocalories (kcal) (3.2–11.8 kcal/min) per 18 holes. This variability was attributed to differences in the methodologies used to assess AEE. Using ecologically valid methods, Kasper et al. (2022) reported some of the lowest AEE values in golf of ∼650–750 kcal per 18‐hole round, with no significant differences across transportation modes (carrying clubs and using a push trolley or electric trolley). Unfortunately, this study did not include a golf cart riding group due to data collection occurring during the COVID‐19 pandemic when golf cart use was not permitted. Although the effect of golf cart use on AEE is debated, no well‐controlled studies have addressed this question. Based on conference proceedings and PhD theses, it has been suggested that golf cart use reduces the AEE of golf from ∼4.4 METs when walking to ∼3.5 METs when riding a golf cart, likely due to a reduction in step count from ∼17,000 to ∼6000 (Murray et al. 2017). However, these findings are based on non–peer‐reviewed data and should be interpreted with caution. There is therefore a need for ecologically valid, controlled studies investigating the physiological impact of golf cart use.

Fatigue, both physical and mental, can impair golf performance influencing swing biomechanics, shot accuracy and decision‐making. Physical fatigue has been shown to negatively affect swing mechanics, reducing shot distance and precision (Higdon et al. 2012), whereas mental fatigue can impair cognitive processes, which are essential for decision‐making, distance judgement and shot execution (Smith et al. 2012; Pan et al. 2025). Evidence suggests that when walking for nine holes (using pushcarts or electric trolleys), participants experienced higher mental focus and physiological load (e.g., AEE) than those riding motorised golf carts (Wolkodoff et al. 2022). However, these potential cognitive benefits may be offset by accumulated fatigue associated with longer rounds, particularly when playing 18 holes or under physically demanding conditions (Smith et al. 2012; Pan et al. 2025). Additionally, fatigue has also been linked to reduced success and shot consistency (Mathers and Grealy 2014). Given that motorised golf carts have been associated with lower AEE (Wolkodoff et al. 2022), their use may help attenuate physical fatigue and in turn maintain mental clarity and physical readiness. Further research is warranted to better understand the trade‐offs between cognitive and physical fatigue across different transportation modes.

In the context of inclusion, golf carts can enable participation for those unable to walk the course and may provide equity for players with higher energy demands, such as those with cerebral palsy (Nardon et al. 2021). However, their use requires careful regulation to ensure that they offer support without providing an unintended competitive edge, for example, golf cart use in tournaments like The G4D Open is strictly monitored, with regulations based on disability or medical exemptions managed by the European Disabled Golf Association (EDGA). Age‐related differences further complicate fairness, as older golfers experience greater metabolic strain, with some spending up to 70% of their round at or above 70% of their maximum heart rate, compared with younger players (Broman et al. 2004). Balancing inclusivity and fairness remains a significant challenge in determining appropriate golf cart use in competitive golf.

Given conflicting reports on the competitive advantage of golf carts, the following question persists: Do golf carts enable more equitable competition or will they introduce a competitive advantage? It is therefore essential to compare walking versus riding a golf cart on physiological (internal load), physical (external load/golf outputs) and cognitive outcomes in competitive settings. The aims of this study were therefore (1) to assess the differences in AEE between competitive golfers walking the course and using a golf cart and (2) to assess the impact of golf cart use on physiological measures, physical performance and cognitive workload. Consistent with our figures and tables, outcomes were prespecified as physiological—AEE, HR, core body temperature and rate of perceived exertion (RPE) and physical—step count and golf outputs (clubhead, ball speed and carry distance), with countermovement (CMJ) metrics (height, net impulse and peak power) taken as a measure of lower‐limb muscle power commonly used to detect fatigue. Cognitive workload was measured using the NASA‐Task Load Index (TLX). To achieve these aims, we recruited 16 high‐level golfers to play two tournament‐style rounds under both conditions. We hypothesised that using a golf cart over 18 holes would result in decreased AEE, HR, core body temperature and RPE; reduce step count without impairing muscle power; improve physical performance (golf metrics); and lower perceived cognitive workload compared with walking the golf course.

## 2 Methods

### 2.1 Participants

Sixteen experienced male golfers (mean ± SD: age 20.9 ± 3.4 years, height: 171.6 ± 45.5 cm, body mass: 78.6 ± 13.8 kg and handicap 2.3 ± 3.7) volunteered to participate in this study. All participants (one tour professional and fifteen amateurs) were current or former students at a specialist golf college (Tournament Golf College, Portugal), with data collection occurring between January and March 2025. Although the study was open to both sexes, only male participants were available at the time of testing, accounting for the single‐sex sample. All participants provided written informed consent prior to enrolment. The study was approved by a local ethics committee and preregistered on ClinicalTrials.gov (ID: NCT06904833).

### 2.2 Experimental Design

In a randomised crossover design, all players completed two rounds of competitive golf on the Championship Faldo Course at Amendoiera Golf Resort, Portugal (18 holes, 6587 m, par 72). Rounds were completed under two different locomotor conditions: (1) walking the course with the bag carried by a caddie (WC) and (2) riding a golf cart driven by a caddie (GC). Golf carts were limited to 10 km per hour and were derestricted, allowing access as close to the putting green as possible. Data were collected over two consecutive days, with comparable weather conditions measured by a handheld wet bulb globe temperature metre (WBGT; AZ Instrument Corp. Taichung City, Taiwan). Players were assigned to the same tee times and four‐balls each day and commenced their rounds between 11:10 and 11:40 a.m., with the mean duration of the rounds consistent between conditions (WC: 5 h, 6 min and GC: 5 h, 5 min). In‐round nutrition was standardised, with players allowed to choose their own foods but requested to consume the same foods for both rounds, whereas fluid intake was *ad libitum* and recorded post‐round. Participants were instructed to avoid strenuous physical activity for 48 h before data collection.

During the rounds, AEE was measured using Actiheart 5 combined heart rate and accelerometery monitors (Camntech, Cambridgeshire, UK), whereas ratings of perceived exertion (RPE), step count (Yamax Digi‐Walker SW650, Shropshire, UK), core body temperature (e‐Celsius Performance Core Temperature Monitoring System, Linton Instrumentation, Norfolk, UK), clubhead speed, ball speed and carry distance (Trackman, Høersholm, Denmark) were measured on the 1st, 6th, 12th and 18th tees. Countermovement jump (CMJ) for measures of height, peak power (PP) and net impulse were assessed via the My Jump smartphone application (Apple Store), pre‐round and immediately post‐round. The NASA‐TLX scale was used to assess perceived workload post‐round. Prior to and after completion of the study, participants were asked whether they believed there was either physical or mental performance advantage of walking the course or riding a golf cart.

### 2.3 Assessment of Activity Energy Expenditure

Preceding each round, participants were fitted with an Actiheart 5 (dimensions: 39.7 × 30.3 × 9.25 mm and weight: 10.5 g). Elastic strapping was used to secure the unit into position though still allowing free movement throughout the golf swing. Participants proceeded to play their round of golf after a ten‐minute signal test was conducted to verify that the R wave of each measurement was accurately recorded, ensuring reliable data collection and minimising the risk of inaccurate readings caused by high noise levels or weak signals. The heart rate (HR) and activity levels were recorded in 15‐s epochs to estimate AEE throughout the rounds. Immediately after each round, the device was removed, and the data were downloaded for subsequent analysis.

### 2.4 Core Body Temperature

Core body temperature was continuously monitored using ingestible telemetry pills. Prior to measurement, each pill was activated with participants ingesting a single calibrated pill at approximately 9 p.m. the evening before each round to ensure sufficient gastrointestinal transit (Ruddock et al. 2014). The pill transmitted real‐time core body temperature data via a wireless telemetry system, which was recorded on the 1st, 6th, 12th and 18th tees (e‐Viewer Performance Monitor, Linton Instrumentation, Norfolk UK). Participants were instructed to follow standard hydration and dietary guidelines to minimise variability in gastrointestinal transit time and data accuracy and wore a yellow wrist band for 48 h after ingestion for identification and safety purposes to ensure that researchers, medical staff or facility personnel were aware that the individual has an ingestible electronic device inside their body.

### 2.5 Ratings of Perceived Exertion

On the 1st, 6th, 12th and 18th tees, participants were verbally requested to state their feelings of perceived exertion using a variation of the traditional Borg RPE scale (Borg and Borg 2002). The Borg CR‐100 RPE scale is proposed to provide greater sensitivity and a more precise assessment of perceived exertion, which ranges from 0 (nothing at all) to 100 (absolute maximum).

### 2.6 Step Count

Pedometers were used to measure participant's step counts. These were reset on the 1st tee prior to the participants first golf shots were taken and then noted on the 6th, 12th and 18th tees. The step count was concluded once the golfer's final putt had been made on the 18^th^ green to obtain a final overall round step count.

### 2.7 NASA‐TLX Scale

Immediately post‐round, perceived workload was assessed using the modified NASA‐TLX 1 to 20 scale, a validated multidimensional tool used to measure across six domains: mental demand, physical demand, temporal demand, performance, effort and frustration level (Hart and Staveland 1988). Players completed the NASA‐TLX, rating each domain on a scale from 1 (low) to 20 (very high), with a higher score indicating a greater perceived workload in that specific domain. Following data collection, scales in each domain were categorised into very high (> 75), high (75–60) and low (< 60) scores, based on guidelines from previous meta‐analyses for global workloads in physical activities that is walking a designated route for comparison between conditions (Grier 2016).

### 2.8 Countermovement Jump

Countermovement jump (CMJ) performance was assessed using the My Jump Lab smartphone application, which has previously been validated for accuracy and reliability in measuring key metrics in applied sport settings (Bishop et al. 2022). Pre‐ and post‐round, each participant completed a standardised 10‐min warm‐up consisting of five minutes of dynamic stretching and practice CMJ trials. Specifically, this involved one set of six repetitions of forward lunges, lateral lunges, the ‘world's greatest stretch’, body mass squats and push‐ups. Participants then performed three practice trials of the CMJ, at 50%, 75% and then 100% of their perceived maximal effort, with hands akimbo throughout the movement. For data collection, all jumps were performed on a flat, wooden decking surface, with all trials filmed on a fourth generation iPad Pro 10 (Apple, London, UK), recording in slow motion at 240 frames per second. The iPad was mounted to a tripod at a height of 0.75 m at 3 m ensuring that full body capture was achieved during the flight phase of the jump. All videos were then analysed using the My Jump Lab app installed on the same iPad Pro device, running on iOS 16.1. Recorded metrics included the following: jump height, peak power (PP) and net impulse. Jump height and PP were computed directly via the app, whereas net impulse was determined by undertaking inverse dynamics calculations. Net impulse was calculated given its previously reported importance as a proxy measure for golf, due to its strong relationship with clubhead speed (Brennan et al. 2023). Regarding the specifics of the inverse dynamic's calculation, take‐off velocity was first determined by computing the square root of jump height multiplied by 2 × gravitational acceleration (9.81 m·s^2^). The resultant take‐off velocity value was then multiplied by each player's body mass to provide a net impulse, as outlined by Wells et al. (2024).

### 2.9 Golf Shot Metrics

Golf shot metrics were collected during both rounds, on 1st, 6th, 12th and 18th tees using a dual‐Doppler radar Trackman 4, which was set up as per the manufacturer's instructions and has been suggested to be the optimal standard of the launch monitor for the sport of golf (Brennan et al. 2024). Specifically, the Trackman was set in *normalised* outdoor mode and positioned 2.5 m behind the ball for all participants, in line with a predetermined target. Recorded metrics included the following: clubhead speed, ball speed and carry distance, which have been shown to have within (Brennan et al. 2023) and between (Bishop et al. 2023) measurement reliability.

### 2.10 Energy and Fluid Intake

Fluid intake was monitored throughout the study. Participants were instructed to consume only the water provided by the research team during each round. Participants received a water bottle containing 1.5 litres (L) of water each day. If additional fluids were required during play, these were supplied by the researchers and their volumes were recorded. At the end of each round, participants returned their water bottles, which were reweighed on portable electronic scales (AccuWeight 201; Nanlgood Network Technology Co. Ltd., Shenzhen, China). The volume of water consumed was calculated by subtracting the final bottle mass from the initial. For consistency, players were instructed to eat the same food pre‐round and during each round. To ensure and monitor compliance, all players recorded their food and fluid intake in the 24 h prior to and during the round using Microsoft Forms. These food diaries were then assessed by a Sport and Exercise Nutrition Register (SENr)–accredited nutritionist with macronutrient composition and energy intake estimated using Nutritics dietary analysis software (Nutritics Ltd, Ireland).

### 2.11 Questionnaire

Participants were instructed to answer a brief questionnaire using Microsoft Forms pre‐ and post‐investigation. The purpose of the questionnaire was to explore participants' beliefs and perceptions regarding the physiological and cognitive advantages or disadvantages of using golf carts versus walking the course and to determine if their views differed towards the two conditions after completing the study.

### 2.12 Statistical Analysis

Descriptive statistics are inclusive of mean ± SD and 95% confidence intervals (95% CI) for environmental and WBGT temperature (°C), fluid intake (L), step count (AU), absolute AEE (kcal), core temperature (°C), HR (beats·min^−1^), CMJ height (cm), CMJ PP (W), CMJ net impulse (N·s^−1^), clubhead and ball speed (mph) and ball carry distance (yards). Responses for RPE and NASA‐TLX measured on ordinal scales were summarised by calculating the frequency of each rating. Ratios and interval data were examined for normality via visual inspections of histogram distributions and Shapiro–Wilk tests, and outliers using boxplots and examination of studentised residuals where appropriate. Within participant comparisons were analysed using paired *t*‐tests, with two‐way repeated measures ANOVAs employed for examination of condition and time effects. Within ANOVAs, sphericity was evaluated using Mauchly's test, and least significant differences post hoc analyses were applied to explore pairwise comparisons. Furthermore, partial eta squared and Hedges' *g* effect sizes (ES) were calculated to assess the practical significance of the findings, with the following thresholds applied: trivial (≤ 0.20), small (0.21–0.60), moderate (0.61–1.20), large (1.21–1.99) and very large (≥ 2.00), in accordance with criteria proposed by Hopkins et al. (2009). Chi‐square tests of independence were conducted to examine differences between post‐round perceptions of the impact of golf cart usage versus walking on internal load and perceived workload domains. All statistical analyses were conducted using SPSS software (version 29 for Windows; SPSS Inc., Chicago, IL, USA), with the alpha level set at *p* ≤ 0.05.

## 3 Results

All data were normally distributed based on histogram examination and Shapiro–Wilk tests (*p* > 0.05), with no outliers highlighted on boxplots and all studentised residuals < ± 3.

Environmental and WBGT on day 1 versus day 2 were 16.6 ± 1.8 versus 17.2 ± 1.0 and 13.0 ± 0.5 versus 13.2 ± 0.7°C, respectively, resulting in a small effect between day 1 and day 2 conditions (environmental: 0.6 ± 1.3°C, *p* = 0.45, 95% CI = −2.6°C–1.5°C, and ES = 0.41 and WBGT: 0.2 ± 0.3, *p* = 0.32, 95% CI = −0.3°C–0.6°C, and ES = 0.32). Although energy and macronutrient intake were replicated between conditions (*p* > 0.05), *ad libitum* fluid intake was significantly greater in the WC (1.6 ± 0.7 L) than the GC (1.2 ± 0.1 L) condition leading to a large effect (0.4 ± 0.6 L, *p* = 0.01, 95% CI = 0.1–0.7 L and ES = 0.80).

**FIGURE 1:**
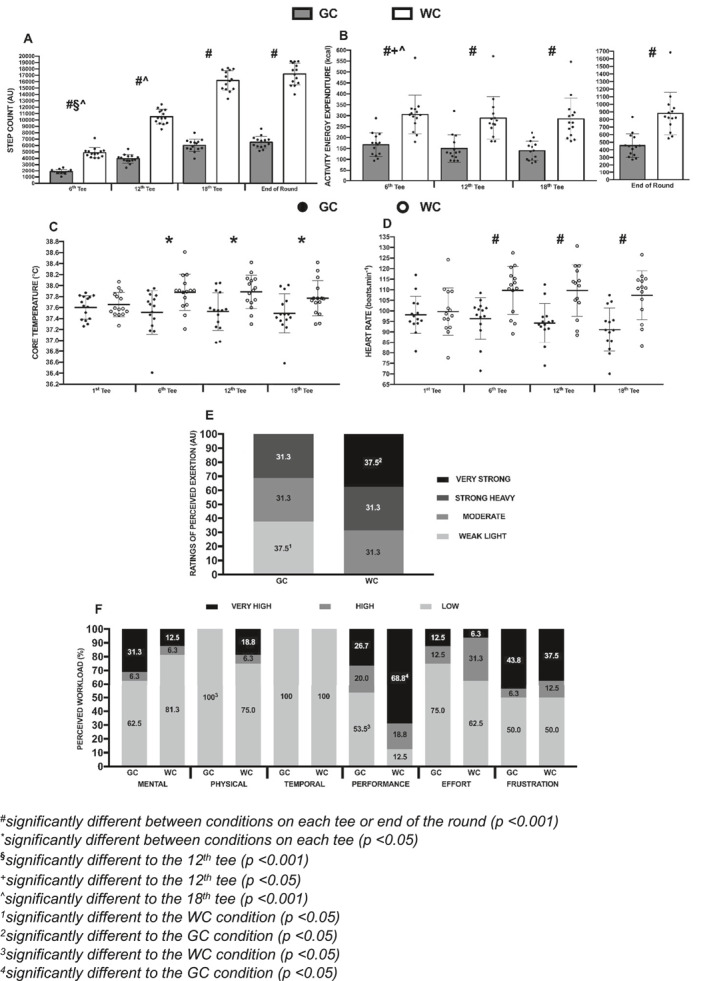
Physical and physiological metrics of step counts, absolute AEE, core temperature, HR and post‐round RPE and NASA‐TLX between the WC and GC conditions.

Step counts were significantly greater in the WC versus GC condition (*p* < 0.001 and ηp^2^ = 0.98) and across tees (*p* < 0.001 and ηp^2^ = 0.99), highlighting an interaction (*p* < 0.001 and ηp^2^ = 0.98) with very large effects among the conditions on the 6th tee (3044 ± 200 AU, *p* < 0.001; 95% CI = 2617–3471 AU and ES = 4.99), 12th tee (6588 ± 274 AU, *p* < 0.001, 95% CI = 6004–7172 AU and ES = 7.07), 18th tee (10126 ± 391 AU, *p* < 0.001, 95% CI = 9293–10959 AU and ES = 7.72) and at the end of the rounds (10670 ± 402 AU, *p* < 0.001, 95% CI = 9813–11527 AU and ES = 7.61) (Figure 1A).

The relative AEE responses were significantly greater in the WC versus GC condition (*p* < 0.001 and ηp^2^ = 0.89) and across tees (*p* < 0.001 and ηp^2^ = 0.74), with an interaction of very large effects among the conditions on the 6th tee (138.04 ± 12.97 kcal, *p* < 0.001, 95% CI = 110.03–166.05 kcal and ES = 7.02), 12th tee (139.28 ± 12.91 kcal, *p* < 0.001, 95% CI = 111.40–167.16 kcal and ES = 6.33), 18th tee (145.89 ± 18.53 kcal, *p* < 0.001, 95% CI = 105.85–185.93 kcal and ES = 7.28) and for total absolute AEE between the rounds (424.06 ± 152.28 kcal, *p* < 0.001, 95% CI = 336.13–511.98 kcal and ES = 7.02) (Figure 1B).

Core body temperature was significantly higher in the WC versus GC condition (*p* < 0.001 and ηp^2^ = 0.51), also demonstrating an interaction (*p* = 0.03 and ηp^2^ = 0.20), with a small effect between WC and GC conditions at the 1st tee (0.05 ± 0.05°C, *p* = 0.31, 95% CI = −0.06°C–0.17°C and ES = 0.28) and significant moderate effects among the 6th tee (0.37 ± 0.12°C, *p* = 0.008, 95% CI = 0.11°C–0.62°C and ES = 1.01), 12th tee (0.36 ± 0.10°C, *p* = 0.003, 95% CI = 0.14°C–0.57°C and ES = 1.09) and 18th tee (0.28 ± 0.10°C, *p* = 0.02, 95% CI = 0.06°C–0.49°C and ES = 0.79) (Figure 1C).

Objective internal load assessments can be seen in Figure 1D. There was a significantly higher HR in the WC than the GC condition (*p* < 0.001; ηp^2^ = 0.83) and an interaction (*p* < 0.001 and ηp^2^ = 0.60), with a trivial effect between WC and GC conditions at the 1^st^ tee (2 ± 2 beats·min^−1^, *p* = 0.47, 95% CI = −3–6 beats·min^−1^ and ES = 0.14) but significant large effects among the 6th tee (16 ± 2 beats·min^−1^, *p* < 0.001, 95% CI = 11–20 beats·min^−1^ and ES = 1.52), 12th tee (15 ± 2 beats·min^−1^, *p* < 0.001, 95% CI = 11–20 beats·min^−1^ and ES = 1.42) and 18^th^ tee (16 ± 2 beats·min^−1^, *p* < 0.001, 95% CI = 11–21 beats·min^−1^ and ES = 1.47).

Figure 1E highlights subjective internal load via assessment of RPE post‐rounds, demonstrating significance between mean scores of the conditions (*p* = 0.04), with 37.5% of participants finding the GC *weak/light*, yet 37.5% found the WC *very strong* (*p* < 0.05).

The post‐round NASA‐TLX scores can be seen in Figure 1. There were no significant differences between the WC and GC conditions for mental (*p* = 0.43), temporal (*p* = 0.31), effort (*p* = 0.41) or frustration (*p* = 0.82) based domains. However, physical demand was perceived to be significant (*p* = 0.01), with 100% of participants scoring *low* in the GC compared with 75% in the WC condition. Performance was also perceived differently between conditions (*p* = 0.03), with 68.6% of participants scoring *very high* in the WC condition compared with 53.3% scoring low in the GC condition (*p* < 0.05).

Performance metrics for clubhead speed, ball speed and ball carry distance can be seen in Table 1. There were no differences in clubhead speeds between the WC and GC conditions (*p* = 0.47 and ηp^2^ = 0.03), though there was a difference in main effect between tees (*p* = 0.003 and ηp^2^ = 0.34), whereby clubhead speeds were significantly faster by small to large effects of 107.6 ± 1.9 mph on the 6th tee in comparison to 105.3 ± 1.7 mph on the 1st tee (2.3 ± 0.7 mph, *p* = 0.003, 95% CI = 0.9–3.6 mph and ES = 1.33) and 106.5 ± 1.9 mph on the 18th tee (1.9 ± 0.3 mph, *p* < 0.001, 95% CI = 0.6–1.8 mph and ES = 0.63), with 107.4 ± 1.7 mph on the 12th tee in comparison to the 1st tee (2.1 ± 0.6 mph, *p* = 0.003, 95% CI = 0.9–3.4 mph and ES = 1.24) and 18th tee (1.0 ± 0.3 mph, *p* = 0.01, 95% CI = 0.3–1.7 mph and ES = 0.50). There were trivial effects between WC and GC conditions (*p* = 0.67; ηp^2^ = 0.01) and across tees (*p* = 0.05; ηp^2^ = 0.19) for ball speeds.

**TABLE 1:** Performance metrics of clubhead and ball speed and carry distance between the WC and GC conditions.

	1st tee	6th tee	12th tee	18th tee
Clubhead speed^a^ ^,^ ^b^ ^,^ ^c^ ^,^ ^d^				
GC	105.5 ± 6.9	107.4 ± 7.6	107.7 ± 6.4	106.5 ± 7.7
WC	105.1 ± 7.0	108.0 ± 7.7	107.2 ± 7.4	106.4 ± 7.6
Ball speed				
GC	155.2 ± 11.3	157.6 ± 10.4	158.9 ± 11.2	156.6 ± 13.1
WC	155.3 ± 10.9	159.2 ± 12.2	157.8 ± 10.9	157.3 ± 11.2
Carry distance^a^ ^,^ ^c^ ^,^ ^e^ ^,^ ^f^				
GC	237.2 ± 22.0	254.4 ± 22.7	249.4^g^ ± 21.5	249.2 ± 27.2
WC	235.8 ± 27.6	257.2 ± 22.9	238.2 ± 30.8	250.4 ± 21.1

Abbreviations: GC, golf cart driven by a caddie; WC, walking the course with bag carried by a caddie.

^a^ Significantly different among combined conditions 1st to 6th tee (*p* < 0.05).

^b^ Significantly different among combined conditions 1st to 12th tee (*p* < 0.05).

^c^ Significantly different among combined conditions 6th to 18th tee (*p* < 0.05).

^d^ Significantly different among combined conditions 12th to 18th tee (*p* < 0.05).

^e^ Significantly different among combined conditions 1st to 18th tee (*p* < 0.05).

^f^ Significantly different among combined conditions 6th to 12th tee (*p* < 0.05).

^g^ Significantly different among GC versus WC conditions (*p* < 0.05).

Although there were trivial effects between the WC and GC conditions for ball carry distance (*p* = 0.49 and ηp^2^ = 0.03), there was a main effect across tees (*p* < 0.001 and ηp^2^ = 0.42), which were significantly further distances by moderate to very large effects of 255.8 ± 5.2 yards on the 6th tee, compared to 236.5 ± 4.3 yards on the 1st tee (19.3 ± 3.1 yards, *p* < 0.001, 95% CI = 12.7–26.0 yards and ES = 4.05), 243.8 ± 6.1 yards on the 12th tee (12.0 ± 3.3 yards, *p* = 0.002, 95% CI = 5.0–19.1 yards and ES = 2.12) and 249.8 ± 5.6 yards on the 18^th^ tee (6.1 ± 2.7 yards, *p* = 0.04, 95% CI = 0.3–11.8 yards and ES = 1.11).

Ball carry distance was greater by a very large effect on the 18th tee than the 1st tee (13.3 ± 4.0 yards, *p* = 0.005, 95% CI = 4.7–21.9 yards and ES = 2.66). Furthermore, there was an interaction (*p* = 0.03 and ηp^2^ = 0.23), with a significant large effect for ball carry distance between WC and GC on the 12th tee (11.3 ± 5.2 yards, *p* = 0.04, 95% CI = 0.2–22.4 yards and ES = 1.70).

Jump metric data collected pre‐ and post‐round can be seen in Table 2. For CMJ height, PP and net impulse, there were significant moderate main effects between the WC and GC conditions (*p* = 0.003 and ηp^2^ = 0.48) and pre‐ to post‐rounds (*p* < 0.001 and ηp^2^ = 0.56). For CMJ height, there was an interaction (*p* = 0.004 and ηp^2^ = 0.46), with a small effect between WC and GC conditions at pre‐round (0.84 ± 0.4 cm, *p* = 0.06, 95% CI = 0.0–1.7 cm and ES = 0.56), yet there was a significant large effect post‐round (2.1 ± 0.5 cm, *p* < 0.001, 95% CI = 1.1–3.0 cm and ES = 1.45). This was subsequently replicated for CMJ PP, with an interaction (*p* = 0.004; ηp^2^ = 0.46) resulting in a moderate effect between WC and GC conditions pre‐round (55.6 ± 26.7 W, *p* = 0.06, 95% CI = −2.6–111.8 W and ES = 0.66) and a significant large effect post‐round (133.2 ± 29.9 W, *p* < 0.001, 95% CI = 69.1–197.3 W and ES = 1.80), with the same trend for CMJ net impulse resulting in a small effect pre‐round (2.1 ± 1.3 N·s^−1^, *p* = 0.13, 95% CI = −0.7–5.0 N·s^−1^ and ES = 0.32) and a significant moderate effect post‐round (6.1 ± 1.3 N·s^−1^, *p* < 0.001, 95% CI = 3.3–8.8 N·s^−1^ and ES = 0.94).

**TABLE 2:** Performance metrics of CMJ height, PP and net impulse between the WC and GC conditions.

	Pre‐round	Post‐round
CMJ height^a^ ^,^ ^b^		
GC	34.3 ± 6.5	33.8 ± 5.6^c^
WC	33.4 ± 5.9	31.7 ± 5.6
CMJ PP^a^ ^,^ ^b^		
GC	2832.8 ± 324.9	2800.7 ± 277.2^c^
WC	2778.2 ± 311.6	2667.4 ± 294.8
CMJ net impulse^a^ ^,^ ^b^		
GC	199.4 ± 25.7	198.1 ± 24.8^c^
WC	197.2 ± 25.9	192.1 ± 25.1

Abbreviations: CMJ, countermovement jump; GC, golf cart driven by a caddie; PP, peak power; WC, walking the course with bag carried by a caddie.

^a^ Significantly different between combined GC and WC conditions (*p* < 0.05).

^b^ Significantly different between combined pre‐ and post‐rounds (*p* < 0.05).

^c^ Significantly different post‐round GC versus WC conditions (*p* < 0.05).

**FIGURE 2:**
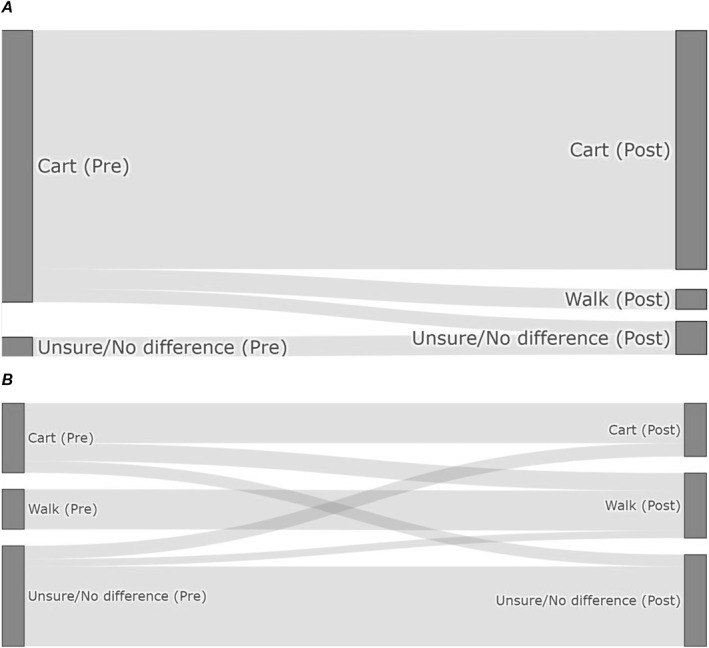
Pre‐ and post‐study belief of the mode of transport most likely to offer a (A) performance and (B) cognitive advantage. Dark grey lines represent number of responses before and after with light grey lines showing the change of decision.

The impact of golf cart usage compared with walking on self‐reported physical (Figure 2A) and cognitive (Figure 2B) advantage was assessed through pre‐ and post‐round questionnaires (*N* = 16). Chi‐square tests of independence revealed no statistically significant differences between expected and observed distributions for both physical (*p* = 0.264) and cognitive (*p* = 0.303) performance between walking and riding a golf cart. However, descriptive analysis revealed notable shifts in participants' perceptions. For physical performance, pre‐round expectations of golf cart impact decreased from 15 to 12 respondents’ post‐round, while walking impact increased from 0 to 2 respondents. Similarly, cognitive performance expectations showed a substantial decrease in perceived golf cart impact (7–3 respondents) and increases in both walking (3–5 respondents) and ‘unsure/no difference’ responses (6–8 respondents).

## 4 Discussion

The aim of the present study was to evaluate differences in physiological, physical and cognitive responses between experienced, skilled golfers walking the course versus riding a golf cart and to examine the potential impact on physical and mental performance indicators. The walking condition resulted in significantly higher AEE, step count, RPE and HR than the golf cart condition. Post‐round CMJ performance was also significantly reduced following walking compared with the golf cart condition. No significant differences were observed in clubhead or ball speed between conditions. These findings indicate that walking during a competitive round of golf imposes greater physiological, physical and cognitive demands, whereas golf cart use may attenuate fatigue, potentially offering a physical advantage.

The present study identified a substantial 424 kcal difference in AEE between conditions, with walking associated with an AEE of 880 kcal and riding a golf cart with an AEE of 456 kcal. A corresponding difference of 10,670 steps was also observed between the two conditions. These values are slightly lower than those reported in previous golf studies, where estimated AEE for 18 holes ranged from approximately 600 kcal with a golf cart to 1200 kcal when walking (Luscombe et al. 2017). The observed discrepancies are likely attributable to methodological differences, as many prior investigations did not employ criterion standard measures of energy expenditure and often failed to subtract the resting metabolic rate (O'Donnell et al. 2024). In the present study, mean AEE was 1.5 kcal·min^−1^ in the golf cart condition and 2.9 kcal·min^−1^ in the walking condition. These findings are consistent with previous research using manual and electric trolleys (Kasper et al. 2022), where AEE ranged from 3.1 to 3.6 kcal·min^−1^. As participants in the current study used a caddie in the walking condition, slightly lower AEE values than previous findings are to be expected.

Step count findings are also in agreement with prior research, where values have ranged from approximately 6000 steps when using a golf cart to 11,000–17,000 when walking the course (Hall et al. 2025). Given that each walking step is estimated to expend ∼0.04 kcal (Wattanapisit and Thanamee 2017), the difference in step counts observed here equates to approximately 428 kcal, which closely aligns with the recorded AEE difference. Based on this assumption, approximately 688 and 261 kcal of the total AEE in the walking and golf cart conditions, respectively, can be attributed to locomotor activity, with the remainder likely due to other golf‐specific movements such as swinging the club. These findings reinforce prior conclusions that locomotion is the primary contributor to total AEE during a round of golf (Kasper et al. 2022). Choosing the more physically active approach to play, such as walking, may confer additional health‐related benefits, including improved cardiovascular fitness, mobility and support for weight management (Bull et al. 2020).

Countermovement jump (CMJ) testing is a widely used method for assessing lower‐body neuromuscular performance, offering a practical and noninvasive indicator of fatigue and recovery status. In the present study, CMJ performance was assessed using the ‘My Jump Lab’ smartphone application, which has demonstrated strong validity and reliability for capturing jump based metrics in applied settings (Haynes et al. 2019). Both time and mode of transportation significantly affected neuromuscular function, with interaction effects suggesting that CMJ height declined to a greater extent following walking than riding a golf cart. Similar patterns were observed for CMJ net impulse and PP, with significant main effects and interactions indicating more pronounced declines in the walking condition (Table 2). These findings suggest that walking induces greater neuromuscular fatigue than golf cart use. Collectively, these results indicate that riding a golf cart may help preserve neuromuscular function and could serve as a practical fatigue management strategy during rounds of golf, such as those encountered in competitive tournament play.

The golf‐specific performance metrics of clubhead speed and ball speed (Table 1) were not significantly affected by mode of transportation, indicating that acute physical exertion associated with walking the course does not appear to impair these aspects of performance over a single 18‐hole round. Small fluctuations in clubhead speed, ball speed and ball carry distance were observed across the round, with clubhead speed and carry distance peaking mid‐round before declining slightly, whereas ball speed showed nonsignificant variability. These findings align with previous work by Langdown et al. (2012), who reported that natural intra‐round variability in swing kinematics is common among skilled golfers and does not necessarily equate to diminished performance. The apparent preservation of golf clubhead speed despite physiological fatigue may be attributed to the explosive, short‐duration nature of the golf swing, which relies on the ability to generate force over brief time periods, an ability that may be maintained even when experiencing mild levels of fatigue. Interestingly, the CMJ also requires rapid force production in short time frames and did exhibit significant reductions in the walking group. As such, this highlights the likely task‐specific nature of how and when acute fatigue may manifest. However, it remains unclear whether such preservation would persist over multiple rounds of play, as in multiday competitive events. It is possible that cumulative fatigue could influence not only gross motor outputs such as swing speed, but also fine motor skills, including distance control and putting accuracy. Further research is warranted to examine these potential effects over extended tournament play and their implications for both physical and technical performance in golf. There was, however, a significant reduction in ball carry distance in the walking group on the 12^th^ tee compared with the golf cart group. The 12^th^ hole at the Amendoiera golf course occurs immediately after a substantial walk up a steep hill (28.14 m ascent), and as such, this reduction in ball carry distance may reflect some acute fatigue in the walking group. As there were no differences in clubhead speed or ball speed between the two groups, this finding is likely to be attributable to changes in ‘shot consistency’ in terms of the quality of the ball strike. Indeed, it has previously been reported that though fatigue has (at best) modest effects on clubhead speed, fatigue can significantly impact shot consistency by as much as 7%–9%, which could result in a reduction in ball flight without any meaningful change in club and ball speed (Higdon et al. 2012). It should be stressed however that this observation was for one metric on one hole and as such should be interpreted with some degree of caution.

Walking the golf course was associated with a greater internal physiological load than using a golf cart, as indicated by consistently elevated HR responses throughout the round. The HR range in the walking condition (83–131 b.m^−1^) exceeded that observed in the golf cart condition (70–112 b.m^−1^), reinforcing the elevated cardiovascular effort required when walking. These findings are consistent with previous research indicating greater HR and energy expenditure during walking (Luscombe et al. 2017). Mean HRs were 109 ± 11 b.m^−1^ in the walking condition and 94 ± 9 b.m^−1^ in the golf cart condition, both of which were higher than those reported previously (Wolkodoff et al. 2022). In the study by Wolkodoff et al. (2022), participants played only nine holes, and the course was relatively flat. In contrast, the current study was conducted over 18 holes on a course with notable elevation changes, including a 28.14 m ascent from the 11th green to the 12th tee. The highest HRs for both conditions were recorded at this point (131 b.m^−1^ walking and 112 b.m^−1^golf cart), highlighting the influence of terrain on internal physiological load. Mean HR values represented approximately 55% (walking) and 47% (golf cart) of participants’ estimated maximum heart rates. According to ACSM guidelines, walking the golf course constitutes moderate physical activity, whereas riding in a golf cart would be classified as a low‐intensity activity (Thompson et al. 2013). These results suggest that walking may not only confer additional cardiovascular health benefits, but also impose a higher internal physiological load, which may affect fatigue and recovery.

A progressive increase in RPE was observed across the round, particularly in the walking condition, indicating an accumulation of physical fatigue (Figure 1E). This was supported by significantly higher post‐round RPE scores in the walking than the golf cart condition. Although elevated RPE did not appear to impair golf‐specific performance metrics, the greater subjective fatigue associated with walking may influence factors such as concentration, recovery and perceived energy, particularly on long or hilly golf courses, in hot and/or humid weather and in multiday or tournament settings or where golfers play over consecutive weeks. Similar associations among increased training loads, RPE and fatigue have been documented in other sports (Pind et al. 2021; Zhao et al. 2023). These findings suggest that though golf cart use may not appear to enhance performance directly, it could serve to reduce fatigue and preserve physical resources across repeated rounds.

Walking the course also imposed significantly greater thermoregulatory strain than riding in a golf cart, as evidenced by higher core temperatures in the walking condition (Figure 1C). This finding aligns with previous research indicating higher metabolic demand during walking (4.3–4.5 METs) than golf cart use (3.5 METs) (Murray et al. 2017). The increased thermoregulatory strain may help explain the significantly greater *ad libitum* fluid intake observed in the walking condition (1.63 ± 0.86 L vs. 1.21 ± 0.44 L), suggesting heightened thirst and underscoring the need for proactive hydration strategies. This also has practical implications, as players walking the course may need to carry, or access, greater volumes of fluid than those riding a golf cart. In a golf‐specific context, both dehydration and elevated core body temperature have the potential to impair physical and cognitive performance. Heat stress may disrupt decision‐making and motor control (Smith et al. 2012), whereas thermal strain and fatigue have been associated with reduced putting accuracy and consistency (Mathers and Grealy 2014). Although these findings suggest a potential benefit of riding a golf cart to mitigate thermoregulatory strain in hot conditions, further golf‐specific research is needed to examine the interaction among hydration status, thermal stress and performance outcomes under competitive, multi‐round conditions. It is biologically plausible that walking the course in cold conditions may be beneficial in helping players to keep warm.

A significantly higher score was observed on the physical demand subscale of the NASA‐TLX, indicating that, unsurprisingly, walking the course was perceived as more physically demanding than riding in a golf cart. However, no significant differences were found for the cognitive subscales, suggesting that the mode of transportation did not meaningfully influence perceived cognitive workload during play. Interestingly, there was a significant difference in the ‘performance’ subscale between conditions with players reporting improved perceptions of ‘performance’ in the walking compared with the golf cart condition. This observation of improved perception of performance when walking compared with riding a golf cart has been suggested previously. For example, Wolkodoff et al. (2022) reported that amateur golfers (mean handicap: 10.9 and mean age: 64 years) improved their mental focus when walking (with an electric trolley or pushcart) compared with riding in a golf cart. These findings raise the possibility that walking may enhance cognitive engagement, and this suggestion should now be explored further in future research.

Prior to and following this study, participants were asked to indicate whether they perceived there to be either a physical or psychological advantage to walking the course, riding a golf cart or neither. No statistically significant differences were found in players' perceptions on the impact of golf cart use versus walking on physical or cognitive performance prior to and following the study. However, descriptive trends from the questionnaires revealed some shifts in perception (Figure 2). Notably, fewer participants reported a perceived physical benefit of cart use after completing the 2 rounds, and a small number of participants began to perceive walking as physically advantageous. This suggests that the actual experience of walking the course may have challenged initial assumptions about its physical demands or benefits and supports the finding of the ‘performance’ subscale of the NASA‐TLX, where players reported a performance preference of walking compared with riding a golf cart. Similarly, perceptions regarding cognitive performance shifted, with fewer participants viewing golf cart use as advantageous. There was a slight increase in those favouring walking or indicating no difference. These findings align with broader themes in the current literature, which suggest that walking may foster greater cognitive engagement during play (Wolkodoff et al. 2022), although empirical support for cognitive enhancement remains inconclusive. The shift in perceptions, despite the absence of statistically significant results, may reflect subtle experiential effects that are not easily captured by objective measures alone. This underscores the value of incorporating subjective assessments into golf performance research, particularly when investigating multidimensional constructs such as fatigue, concentration and perceived effort. Further research with larger sample sizes and more sensitive psychometric tools are now warranted.

Although this study offers valuable insights into the existing literature and addresses timely questions within the sport of golf, several limitations must be acknowledged, many of which are inherent to the challenges of conducting research in real‐world environments (Close et al. 2019). Each transportation method was tested over a single round of golf, whereas tournament play typically spans three to 4 days. As such, the cumulative effects of fatigue, hydration demands and neuromuscular load over multiple rounds remain unknown. Although weather conditions were stable during the testing period, the results may not be fully generalisable to competitive play in more diverse climates, particularly in hot and humid environments exceeding 30°C. Conversely, future studies should also consider colder conditions, where the use of golf carts may offer different physiological or performance‐related effects. Although the study was open to both sexes, no female players were available during the testing period due to gender imbalance within the golf college where participants were recruited. This limits the generalisability of findings and highlights the need for future research to examine potential sex‐based differences in physiological and cognitive responses to golf‐specific exertion. Moreover, while the inclusion of professional golfers during tournament play would have enhanced the ecological validity of findings, the use of golf carts is prohibited in most professional tournaments, precluding the feasibility of such a study within that context. Finally, the inclusion of older golfers and those with disabilities is warranted to ensure greater representativeness of the broader golfing population.

## 5 Conclusion

This study is the first to report differences among physiological, physical, and cognitive responses between walking the course and using a golf cart across a full 18‐hole round under simulated tournament conditions with high‐standard golfers. Walking the course resulted in significantly greater AEE and step counts, accompanied by higher heart rates, core body temperature, and *ad libitum* fluid intake‐findings that may have implications for fuelling and hydration strategies during competition. NASA‐TLX and RPE scores suggested that the physical demand was reduced using a golf cart, thus indicating that use of this transport mode may help mitigate against physical fatigue. Post‐round reductions in neuromuscular performance, as evidenced by CMJ metrics, were more pronounced following walking, indicating increased fatigue. There were no significant effects of transport for the golf‐specific performance outcomes of clubhead and speed although ball carry distance was reduced on the 12^th^ tee. These findings suggest that walking imposes a greater physiological load in skilled players over a single round. Future research is warranted to examine whether these physiological and perceived fatigue responses translate into meaningful competitive outcomes, particularly over multiple rounds of play. Additionally, studies should aim to include female participants to explore potential sex‐based differences, as well as golfers with disabilities and older athletes to enhance the applicability of findings across the broad golfing population.

## Funding

The authors would like to thank DP World Tour, The R&A and Ladies European Tour for their financial support of this research.

## Ethics Statement

The study was approved by the Liverpool John Moores University Ethics Committee (reference: 24/SPS/075) and preregistered on ClinicalTrials.gov (ID: NCT06904833).

## Conflicts of Interest

GC performs nutrition consultancy for DP World Tour Golf and AOD for Ladies European Tour, whilst AM is the chief medical and scientific officer for DP World Tour Golf. The other authors report no conflicts of interest with the content of this article.
